# Evaluation of the simulator with automatic irrigation control system designed for countermeasures of internal contamination in dental unit water lines

**DOI:** 10.1016/j.heliyon.2020.e04132

**Published:** 2020-06-10

**Authors:** Keisuke Okubo, Takashi Ito, Kentaro Okamoto, Ichiro Yamamoto, Hajime Mizutani, Yusuke Kawata, Yasuyoshi Shiota, Masahiro Ito, Shin Nakamura, Masako Tai, Tadashi Yamamoto, Shogo Takashiba

**Affiliations:** a Department of Pathophysiology - Periodontal Science, Okayama University Graduate School of Medicine, Dentistry and Pharmaceutical Sciences, 2-5-1 Shikata-cho, Kita-ku, Okayama 700-8558, Japan; b Division of Dentistry, Tottori Municipal Hospital, 1-1 Matoba Tottori, Tottori 680-0501, Japan; c Center for Innovative Clinical Medicine, Okayama University Hospital, 2-5-1 Shikata-cho, Kita-ku, Okayama 700-8558, Japan; d Dental Department Marketing Division, TAKARA BELMONT Corporation, 2-1-1 Higashishinsaibashi, Chuo-ku, Osaka 542-0083, Japan; e Research and Development Department, TAKARA BELMONT Corporation, 2-1-1 Higashishinsaibashi, Chuo-ku, Osaka 542-0083, Japan; f Department of Periodontics and Endodontics, Okayama University Hospital, 2-5-1 Shikata-cho, Kita-ku, Okayama 700-8558, Japan

**Keywords:** Microbiology, Biomedical devices, Safety engineering, Microorganism, Biofilms, Dentistry, Dental chair unit water line (DUWL), Automated simulator, Water decontamination

## Abstract

The prevention of nosocomial infections is an imperative task. The dental chair unit (DCU) is an indispensable device used in dental treatment. However, it is known that the dental unit water line (DUWL) can become contaminated with biofilm, consisting mainly of heterotrophic bacteria (HB). Recently, the International Organization for Standardization specified the methods for testing DUWL contamination management. On these grounds, a simulator reproducing DUWL was prepared to standardize the examination method of the DUWL contamination.

**Objectives:**

To evaluate the reproducibility of the DUWL simulator, monitor the DUWL contamination states, and test the efficacy of a commercial decontaminant for DUWL.

**Methods:**

The DUWL simulator was assembled by a DCU manufacturing company. The simulator's DUWL was filled with tap water (TW), and left for approximately one year. Neutral electrolyzed water (NEW) was used as a decontaminant for DUWL. Both TW and NEW were passed through DUWL in a timely manner simulating daily dental treatment. Water was sampled from the air turbine hand piece weekly for 4 weeks and used for HB culture. Contamination status was evaluated by measuring bacterial adenosine triphosphate release and by culturing on Reasoner's 2A medium.

**Results:**

The DUWL released contaminated water had a bacterial count of over 6 × 10^4^ cfu/mL. After passing NEW through DUWL for 1 week, the count drastically decreased to its basal level and remained steady for 4 weeks. However, TW showed no effect on DUWL decontamination throughout the examination periods.

**Conclusions:**

The DUWL simulator could be useful to examine the efficacy of the decontaminant for DUWL and development of new methods in DUWL contamination management.

## 1 Introduction

In recent years, the prevention of nosocomial infections in elderly people and compromised hosts has been recognized as a serious issue. Although the dental unit is an essential instrument to perform dental treatments, it has been suggested that the water coming through the dental unit water line (DUWL) is already contaminated with heterotrophic bacteria (HB) [1] and is the cause of contamination in dental clinics [2, 3, 4]. Opportunistic pathogens such as *Pseudomonas* and *Acinetobacter* were detected in the dental unit water [5]. Biofilm formation on the inner side of narrow DUWL mainly consists of HB that are maturing over time [1].

Despite these issues, the global guidelines for countermeasures have not been established yet, as there are few reports that show direct clinical evidence for the infection by DUWL. In the meantime, the American Dental Association has established a guideline specifying that the number of HB should be less than 500 cfu/mL in DUWL water [6]. Furthermore, International Organization for Standardization (ISO) has established the test method (ISO16954) for control of contamination of DUWL in 2015 [7]. However, it is extremely difficult to comply with these ISO Standards, which are strictly defined in terms of management of test water, microorganisms, and test equipment.

On the other hand, there have already been several studies about biofilm formation using either DUWLs of active dental chair unit or unique model [8, 9, 10, 11, 12]. In addition decontamination methods for water pollution have been reported, not only in the field of dentistry but also in the research field on general water pipes [13, 14]. However, we contemplated that it was necessary to examine under a standardized environment to quantify the water pollution in DUWL which has complicated and unique structures. Therefore, we considered that the actual conditions of DUWL in clinical environments should be simulated by an established analysis system. In this study, we constructed the automatic irrigation control simulator which reproduced DUWL conditions and examined the efficacy of decontaminant for DUWL in standardized environment.

## 2 Materials and methods

### 2.1 Test equipment

**Figure 1:**
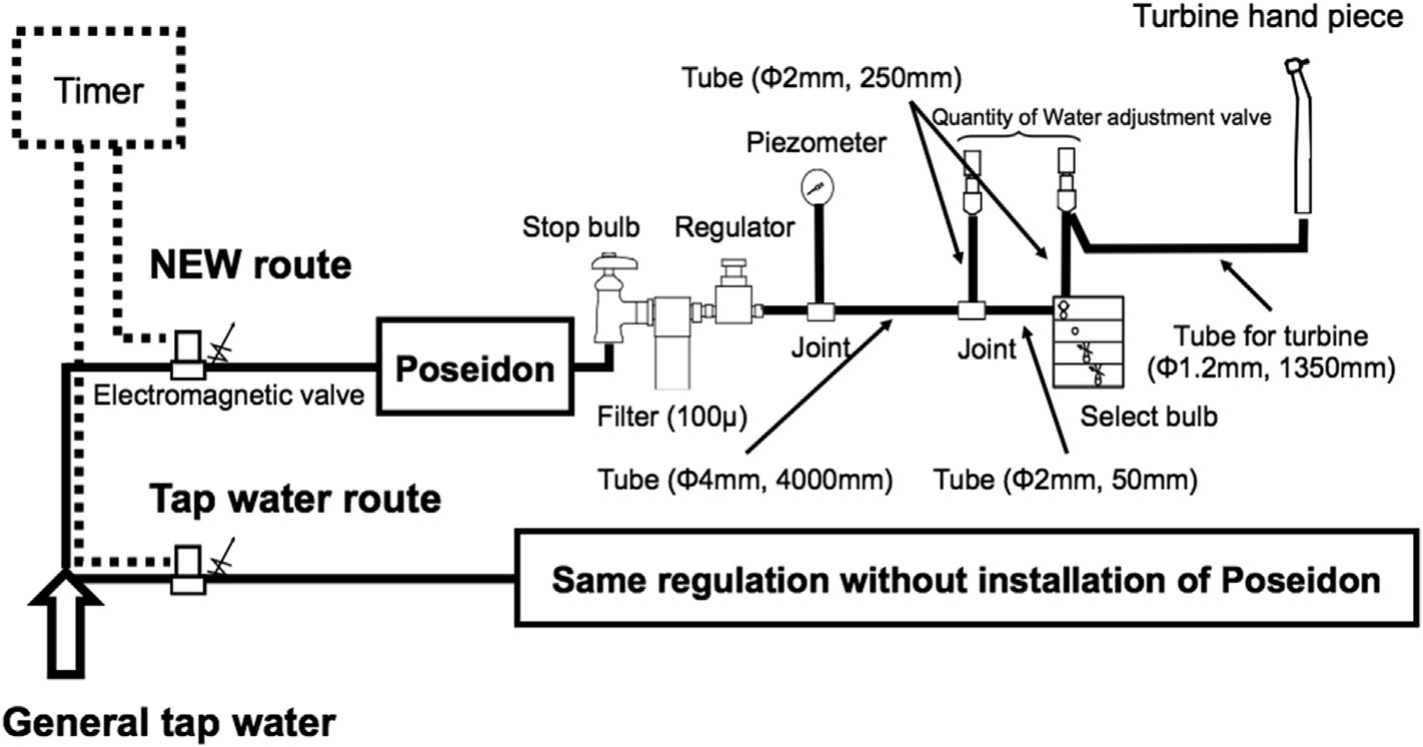
The design of DUWL simulator (Schematic view). General flow of TW through both supply lines. Passage of water is controlled by the electromagnetic valves, interlocked with timer. The NEW route is same as TW route except for a built-in-Poseidon. This design simulated the commercial model of a dental chair unit.

The dental unit simulator was assembled with the co-operation from TAKARA BELMOMT Corp. (Osaka, Japan) as shown in Figure 1. This simulator mimicked the designs of dental chair units which are manufactured by TAKARA BELMOMT Corp. Water pressure in DUWL was set to 0.2 MPa monitored by piezometer in the simulator. Information other than those indicated in this paper must be kept confidential as requested by TAKARA BELMOMT Corp. The automatic irrigation control program was set up according to ISO16954 as far as possible with time settings reflecting actual conditions in general dental clinics (Table 1), which was the result of surveys performed by TAKARA BELMOMT Corp. using their products. This study was performed for four weeks using this simulator in which tap water (described below) was retained at 20 °C for approximately one year.

**Table 1:** Time setting of the automatic irrigation control system for air turbine.

Time	Condition	Time	Condition
7:59	Run	12:00	Run
8:00–8:29	Suspension	12:01–13:59	Suspension
8:30	Run	14:00	Run
8:31–8:59	Suspension	14:01–14:29	Suspension
9:00	Run	14:30	Run
9:01–9:29	Suspension	14:31–14:59	Suspension
9:30	Run	15:00	Run
9:31–9:59	Suspension	15:01–15:29	Suspension
10:00	Run	15:30	Run
10:01–10:29	Suspension	15:31–15:59	Suspension
10:30	Run	16:00	Run
10:31–10:59	Suspension	16:01–16:29	Suspension
11:00	Run	16:30	Run
11:01–11:29	Suspension	16:31–16:59	Suspension
11:30	Run	17:00	Run
11:31–11:59	Suspension	17:01–7:58	Suspension for night

Total run: for 16 min.

### 2.2 Water

Domestic tap water (TW; Water Works Bureau at Okayama City, Japan) was used for the experiment. Neutral electrolyzed water (NEW) was generated using the device Poseidon-S (Self Medical Corp., Kyoto, Japan) [8]. This system has a sensor that detects the flow of water and generates chlorine from the chloride ions automatically through an electric current. Chlorine reacts with water immediately forming hypochlorite ions and hypochlorous acid. These products have strong bactericidal effects [15, 16]. This system can adjust residual chlorine concentration up to approximately 7.0 ppm in NEW and the flow rate is approximately 3 L/min. Fresh TW was used as control. Water sample was collected from the turbine hand-piece weekly for 4 weeks.

### 2.3 Bacterial examination

HB were examined in 100 μL of test water directly collected from DUWL, or 100 μL of liquid culture sample. The culture sample was obtained by inoculating 50 mL of test water collected from DUWL into 450 mL of Reasoner's 2A (R2A) liquid medium [17] (Wako Pure Chemical Industries, Ltd., Osaka, Japan) and cultured at 20 °C for 6 days. Bacterial growth analysis was performed by the following method.

#### 2.3.1 Measurement of the amount of adenosine triphosphate (ATP)

Bacterial growth activity was determined by the amount of ATP in the bacteria. ATP quantity was measured using the Lucifer HS kit and Lumi-tester C-110 (Kikkoman Bio-Chemiphar, Tokyo, Japan), which employs luciferin-luciferase reaction [18]. The amount of luminescence was recorded as the values of relative light unit (RLU, detection range: 1.0 × 10^−16^ to 3.0 × 10^−11^ mol of ATP). Residual ATP was removed by adenosine phosphate deaminase. The concrete operation was performed according to the manufacturer's instruction included in the kit.

#### 2.3.2 Counting number of colonies

To determine the colony count, 100 μL of test water was diluted at 10^5^ times with phosphate buffered saline. This was then inoculated and cultured on Reasoner's 2A (R2A) agar medium [17] (Becton, Dickinson and Company, Tokyo, Japan) at 20 °C for 6 days.

### 2.4 Statistical analysis

Statistical processing was performed using Student's *t*-test (Microsoft Excel) after using an F-test (Microsoft Excel) to analyze the variance between groups. *P*-values less than 0.05 were considered statistically significant.

## 3 Results

### 3.1 Changes of ATP amount in DUWL water

**Figure 2:**
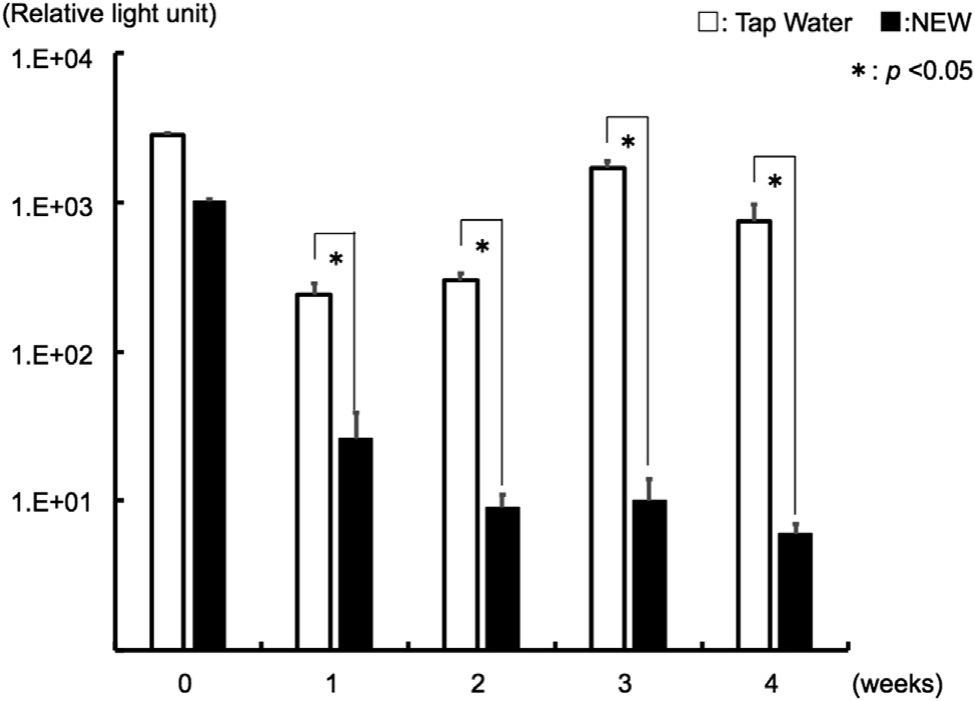
Chronological changes of ATP amount. The samples (100 μL of TW and NEW) were corrected from DUWL every week from the 0-week to 4-weeks. The ATP amount was measured by luciferin-luciferase reaction method. Error bars indicate standard deviation. Measurements were performed in triplicates. *p*∗<0.05.

Each sample (100 μL of TW and NEW) collected from DUWL at the week 0, showed the ATP amounts of microorganisms to be well over 10^3^ RLU, whereas the ATP quantity of control water (fresh TW) was 8 RLU (data not shown). Although the ATP quantity of TW was stable at 10^2^-10^3^ RLU, the ATP quantity of NEW was significantly reduced compared to TW in all examined samples for 4 weeks (Figure 2).

### 3.2 Changes of ATP amount in liquid culture of DUWL water

**Figure 3:**
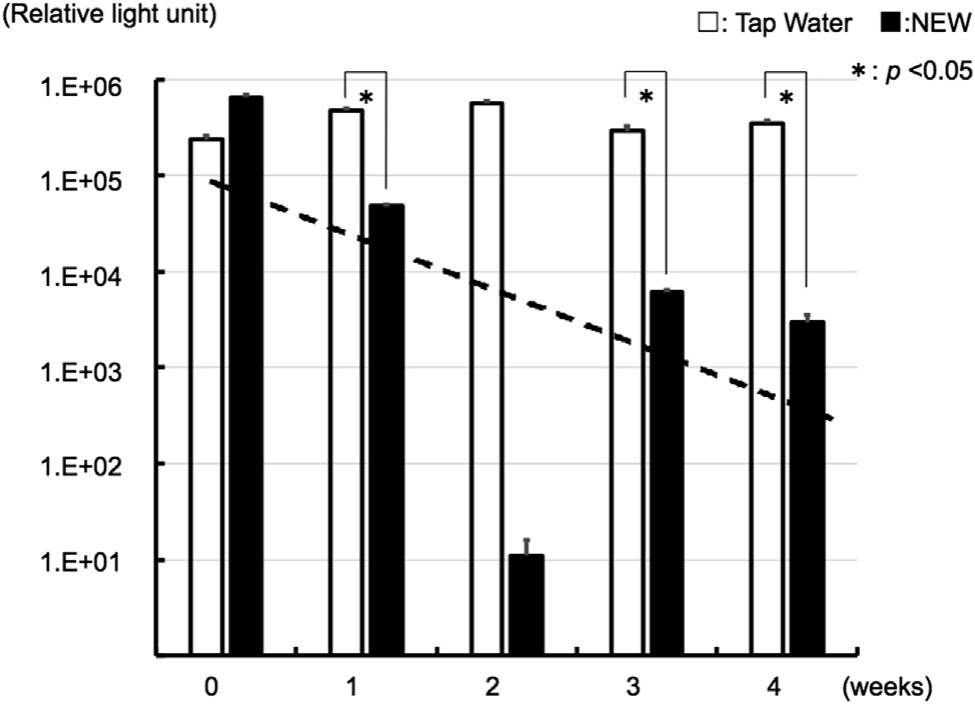
Chronological changes of ATP amount after culture in liquid medium. The sample (TW and NEW) was liquid-cultured for 6 days after correction from DUWL from the 0-week to 4-weeks. The ATP amount was measured by luciferin-luciferase reaction method. The dotted line is exponential approximation line of ATP in NEW (y = 273615e^−1.287x^, R^2^ = 0.2488). Error bars indicate standard deviation. Measurements were performed ten times, *p*∗<0.05.

Each sample (TW and NEW) was liquid-cultured for 6 days after collection from DUWL at week 0, with ATP amounts over 10^5^ RLU. Although the ATP quantity of TW remained stable over 10^5^ RLU (the average ATP value was 109,543), the ATP quantity in NEW decreased significantly compared to TW in a time dependent manner, except for the sample collected at week 2. The exponential approximation line for ATP in NEW was calculated (y = 273615e^−1.287x^, R^2^ = 0.2488; the data at week 2 was excluded) (Figure 3).

### 3.3 Changes of bacterial colonies on the agar culture of DUWL water

**Figure 4:**
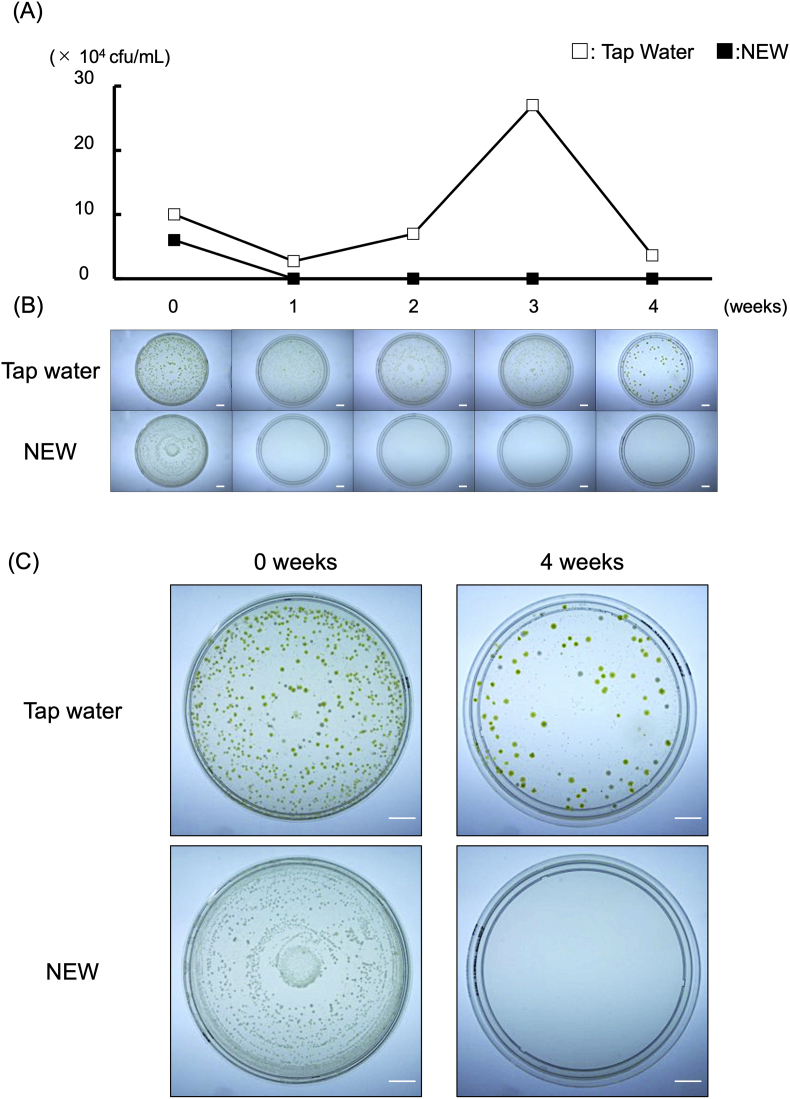
Bacterial findings on the agar medium. (A) Chronological changes of the number of living bacteria density. Each sample (TW: □ and NEW: ■ was agar-cultured for 6 days after collection from DUWL. Colony count was performed and the average of three plates are shown. (B) Macroscopic analysis of microbial colonies. The typical images are shown for each sample. Upper panels: TW, Lower panels: NEW Scale bar: 1 cm. (C) Enlarged images of representative plates. These enlarged images are representative in TW and NEW plates collected at 0 and 4 weeks. Scale bar: 1 cm.

For each sample (TW and NEW), agar-cultured for 6 days after collection from DUWL at week 0, the colony counts were over 10^4^ cfu/mL. No living bacteria were detected in control water (fresh TW; data not shown). The observation of microbial colonies indicated different properties; yellow colonies were abundant in TW, whereas white colonies predominated in NEW. Although the colony count of TW was over 10^4^ cfu/mL, no colonies were identified in NEW sample collected from week 1 to week 4, with values below the detection limit. In TW sample collected at week 4, yellow colonies were still observed, along with the sporadic appearance of red colonies (Figure 4).

## 4 Discussion

The purpose of the DUWL simulator is to compare several contamination control protocols in a standard environment. In this study, the validity of the simulator was evaluated by monitoring the level of contamination and verifying the efficacy of NEW, a commercial decontaminant for DUWL. The simulator represented a standard setting which imitated the conditions in the dental clinic and was compatible with ISO standards. The advantage of this simulator is that we assembled the same structure as DUWL used in the dental clinic precisely, with cooperation of a dental chair unit manufacturer, TAKARA BELMONT Corp. This assembly makes it possible to perform examinations under actual conditions and has excellent reproducibility. In addition, the test method is based on ISO standards as much as possible, so that it has objectivity and potential for future development.

At the start of this experiment, test water was stored in DUWL for one year for microbial propagation. As a result, the number of live bacteria in DUWL simulator increased above 10^4^ cfu/mL. As a previous study reported contamination at and above 10^4^ cfu/mL in DUWL [3], we reasoned that the test water was appropriate for examination of contamination in the DUWL simulator.

In TW, microbial contamination was constantly detected throughout the examination period. The contamination didn't reduce even if DUWL was irrigated by flushing with fresh TW. This result suggested that it is difficult to reduce microbial contamination once it occurs inside DUWL.

Bactericidal action of NEW is evident by the powerful oxidation potential of hypochlorous acid [15, 16] which is produced by electrolysis of water. After 1 week of irrigation with NEW, the contamination of DUWL was significantly reduced as compared to irrigation with fresh TW as shown by ATP analysis. The contamination quantity was observed to be the same as of control water (fresh TW) during the examination periods. Although further analysis is required, it is suggested that the percentage of HB was also significantly reduced by NEW irrigation.

TW primarily formed yellow or red colonies on agar medium. According to a previous report [19], it is speculated that the yellow colonies belong to *Sphingomonas paucimobilis* and the red colonies belong to *Methylobacterium mesophilicum*. On the contrary, white colonies predominated in NEW initially. It was assumed that these colonies were from *Acinetobacter haemolytics* [19]. This result suggests that the HB flora in DUWL was affected by the chemicals. Furthermore, no visible colonies were observed in NEW after the first week; however, ATP quantities showed an increase after culture in the medium. This result suggests the existence of microorganisms which could not grow on R2A agar medium. Therefore, examination of detailed microbial flora for a prolonged period will be necessary in future.

The simulator can be disassembled into components. Therefore, it is possible to examine the changes in growth and characteristics of microbial biofilm in each part. In other words, by analyzing the biofilm formed on each component of this simulator, it is possible to compare the changes of composition and amounts of bacteria in each of the DUWL components under the same environment. In future, it may be useful to evaluate the effectiveness of other functional waters or antibacterial materials and to utilize the various contamination control methods in clinical dentistry.

## 5 Conclusion

The simulator designed and assembled in this study is suitable to verify the contamination in DUWL and examine various contamination control protocols in clinical conditions.

## Declarations

### Author contribution statement

Keisuke Okubo: Conceived and designed the experiments; Performed the experiments; Analyzed and interpreted the data; Contributed reagents, materials, analysis tools or data; Wrote the paper.

Takashi Ito, Shogo Takashiba: Conceived and designed the experiments; Analyzed and interpreted the data; Wrote the paper.

Kentaro Okamoto, Masahiro Ito, Shin Nakamura, Masako Tai: Contributed reagents, materials, analysis tools or data.

Ichiro Yamamoto, Hajime Mizutani: Conceived and designed the experiments; Contributed reagents, materials, analysis tools or data.

Yusuke Kawata, Yasuyoshi Shiota: Conceived and designed the experiments.

Tadashi Yamamoto: Analyzed and interpreted the data; Wrote the paper.

### Funding statement

This research did not receive any specific grant from funding agencies in the public, commercial, or not-for-profit sectors.

### Competing interest statement

The authors declare no conflict of interest.

### Additional information

No additional information is available for this paper.
